# Off-label use of rilpivirine in combination with emtricitabine and tenofovir in HIV-1-infected pediatric patients: A multicenter study

**DOI:** 10.1097/MD.0000000000003842

**Published:** 2016-06-17

**Authors:** Lola Falcon-Neyra, Claudia Palladino, María Luisa Navarro Gómez, Pere Soler-Palacín, María Isabel González-Tomé, Santiago J. De Ory, Marie Antoinette Frick, Clàudia Fortuny, Antoni Noguera-Julian, Elena Bermúdez Moreno, Juan Luis Santos, Peter Olbrich, Luis F. López-Cortés, Verónica Briz, Olaf Neth

**Affiliations:** a Unidad de Enfermedades Infecciosas e Inmunopatologias, Hospital Infantil Virgen del Rocío, Instituto de Biomedicina de Sevilla (IBiS), Seville, Spain; b Research Institute for Medicines (iMed.ULisboa), Faculty of Pharmacy, University of Lisbon, Lisbon, Portugal; c Sección de Enfermedades Infecciosas, Servicio de Pediatría, Hospital General Universitario Gregorio Marañón, Madrid; d Unitat de Patologia Infecciosa i Immunodeficiències de Pediatria, Hospital Universitari Vall d’Hebron, Institut de Recerca Vall d’Hebron, Universitat Autònoma de Barcelona, Barcelona; e Servicio de Infecciosas Pediátricas, Hospital Universitario Doce de Octubre; f Laboratorio InmunoBiología Molecular, Hospital General Universitario Gregorio Marañón. Instituto de Investigación Sanitaria Gregorio Marañón (IiSGM); Networking Research Center on Bioengineering, Biomaterials and Nanomedicine (CIBER-BBN), Madrid; g Unitat d’Infectologia, Servei de Pediatria; Hospital Sant Joan de Déu, Universitat de Barcelona, Barcelona, Spain; h Servicio de Enfermedades Infecciosas, Hospital General Universitario Gregorio Marañón, Madrid; i Unidad de Enfermedades Infecciosas e Inmunodeficiencias, Sección Urgencias de Pediatría, Hospital Universitario Virgen de las Nieves, Granada; j Enfermedades Infecciosas, Microbiología y Medicina Preventiva. Instituto de Biomedicina de Sevilla/Hospital Universitario Virgen del Rocío/CSIC/Universidad de Sevilla, Seville; k Unit of Viral Infection and Immunity, National Center for Microbiology, Institute of Health Carlos III, Majadahonda, Madrid, Spain.

**Keywords:** adolescents, antiretroviral therapy, children, HIV-1, rilpivirine

## Abstract

To assess the safety and efficacy of rilpivirine in combination with emtricitabine and tenofovir (RPV/FTC/TDF) as a once-daily single-tablet regimen (STR) in HIV-1-infected children and adolescents we performed a multicenter case series study of HIV-1-infected patients. Inclusion criteria were initiation of therapy with RPV/FTC/TDF before the age of 18. Patients were divided into undetectable viral load (uVL) group, HIV-1 RNA < 20 copies/mL on stable combined antiretroviral therapy (cART), and detectable viral load (dVL) group, HIV-1 RNA ≥ 20 copies/mL at RPV/FTC/TDF initiation. Patients were monitored from the date of RPV/FTC/TDF initiation until June 30, 2015, RPV/FTC/TDF discontinuation or failure to follow-up. Seventeen patients (8 in uVL and 9 in dVL group) with age between 11.6 and 17.6 were included. Reasons for switching were toxicity (n = 4) and simplification (n = 4) in uVL; viral failure (n = 8) and cART initiation (n = 1) in the dVL group. After a median follow-up of 90 (uVL) and 40 weeks (dVL), 7/8 (86%) patients maintained and 8/9 (89%) achieved and maintained HIV-1 suppression. Median CD4 count increased from 542 to 780/μL (uVL, *P* = 0.069) and 480 to 830/μL (dVL, *P* = 0.051). Five patients (2 in uVL and 3 in dVL) improved their immunological status from moderate to no immunosuppression. Serum lipid profiles improved in both groups; cholesterol dropped significantly in the dVL group (*P* = 0.008). Grade 1 laboratory adverse events (AEs) were observed in 3 patients. No clinical AEs occurred. Adherence was complete in 9 patients (5 in uVL and 4 in dVL); 1 adolescent interrupted treatment. Once-daily STR with RPV/FTC/TDF may be a safe and effective choice in selected HIV-1-infected adolescents and children.

## 1 Introduction

The management of HIV-infected pediatric patients is a challenge due to the complexity of their social environment, the desire to be accepted by their peers, and poor knowledge and a negative attitude in relation to their HIV status. Furthermore, lack of adherence (particularly in adolescents), high pill burden, inadequate support, substance abuse, few age-appropriate formulations to ensure appropriate drug exposure, and differences in pharmacokinetics between children and adults impact efficacy and safety.^[1–6]^

In adults, single-tablet regimens (STR) are associated with high adherence rates and improvement of quality of life.^[7]^ STR also avoid selective nonadherence—not taking all components of a regimen.^[8]^ Rilpivirine (TMC278), a new non-nucleoside reverse transcriptase inhibitor (NNRTI), is available as a single-agent tablet or in combination with FTC plus TDF in an STR. Several studies have indicated RPV/FTC/TDF STR being a favorable choice in naïve and selected treatment-experienced HIV-1-infected adults.^[9–12]^ This STR has been approved by the FDA in naïve HIV-1-infected adults with HIV-1 RNA ≤100,000 copies/mL or virologically suppressed (HIV-1 RNA < 50 copies/mL) adults with no mutations associated with resistance to NNRTIs, FTC, or TDF.^[13]^

RPV has been approved in the United States for children age ≥12 years, but not in Europe, and data on its efficacy in this age group is very limited to date. PAINT (Pediatric study in Adolescents Investigating a new NNRTI TMC278) is a multicenter ongoing trial evaluating the pharmacokinetics, safety, and efficacy of RPV in treatment-experienced and naïve HIV-1-infected adolescents age 12 to 18 years.^[14,15]^ Here, we present long-term data from children and adolescents with perinatally acquired HIV-1 infection treated with off-label RPV/FTC/TDF.

### 1.1 Study patients and methods

In this multicenter observational study, 17 vertically HIV-1-infected children and adolescents (<18 years) were enrolled from March 2013 from 6 pediatric reference hospitals (Cohort of the Spanish Pediatric HIV Network, CoRISpe,^[16]^ see Annex). STR with RPV/FTC/TDF (25/200/245 mg) was prescribed under the Spanish off-label medication use program.^[17]^ Individuals were monitored from baseline (date of RPV/FTC/TDF initiation) until June 30, 2015 (administrative censoring date), RPV/FTC/TDF discontinuation or failure to follow up. Demographic, clinical, and laboratory parameters were extracted from the CoRISpe database. Adherence was evaluated by pill count, assessing the dose taken, and interviewing parents/guardians, and was summarized as a single percentage for each combined antiretroviral therapy (cART) regimen for each patient and categorized as poor (<70%), intermediate (70–90%), good (91–99%), or perfect (100%). Immunological category and clinical stage were based on the CDC classification.^[18]^ Major mutations from the IAS-USA mutation list 2013 were used to define resistance to RPV, TDF, and/or FTC.^[19]^ Plasma VL (HIV-1 RNA) was measured by Amplicor Monitor assay (Roche Diagnostic Systems, Pleasanton, CA, USA) and real-time NASBA (Easy Mag y Nuclisens Easy Q, BioMerieux, Boston, MA, USA) with a detection limit of 20 HIV-1 RNA copies/mL. Virological failure was defined as the inability to achieve or maintain suppression of viral replication below 20 copies/mL. CD4 were quantified by flow cytometry (Beckman Coulter FC-500 Cytometer, Beckman Coulter, Inc., Brea, CA, USA and Becton-Dickinson-FACScalibur, Franklin Lakes, NJ, USA). Toxicity was monitored every 6 months during follow-up. Parameters monitored included renal toxicity, hepatic toxicity, and laboratory parameters such as lipid profile cholesterol and glucose. Rash, dizziness/light-headedness, insomnia or abnormal dreams, headaches, nausea, fatigue, and depression were also evaluated. Adverse events (AEs) severity was graded using the Division of AIDS AEs grading table.^[20]^ Ethical approval was obtained from the ethical committees of all hospitals.

Quantitative variables were summarized using medians and interquartile ranges (IQRs), whereas absolute values and relative frequencies were used for qualitative variables. Demographic, clinical, and laboratory variables were analyzed according to baseline VL: patients with undetectable VL (uVL) and patients with detectable viral load (dVL). Continuous variables were compared between groups using the Mann–Whitney *U* test. The nonparametric Wilcoxon signed-rank test was applied to determine differences for measurements at different points in time. The differences were considered statistically significant for *P* values <0.05. The statistical analyses were performed using SPSS software (v. 19.0, Chicago, IL).

## 2 Results

Seventeen subjects were included in the study. Demographic, clinical, and laboratory baseline characteristics are summarized in Table 1. Two were children age 11.6 and 11.7 years and 15 were adolescents age 16.7 years (IQR: 15.8–17.3). Ten were girls (59%) and 13 (76%) Caucasian. At time of enrolment 7 (41%) subjects presented moderate immunosuppression and 2 (12%) had a clinical stage C. At baseline all patients showed HIV-1 RNA <10,000 copies/mL. At the start of the RPV-based regimen, 1 patient was cART-naïve and the rest had been exposed to cART for a median of 10.0 (IQR: 7.6–12.2) years. Four were on an NNRTI and 12 on a protease inhibitor-based regimen. Five adolescents had accumulated reverse transcriptase resistance-associated mutations (RAMs): in the uVL group, 1 patient had the M184V mutation, 1 individual the M184V and G190A mutations, and 1 subject the T215Y and M41L mutations. In the dVL group, 1 patient had the T215Y mutation and 1 the T215Y and Y181C mutations; the latter reduces susceptibility to RPV 3-fold (Table 1).

**Table 1:**
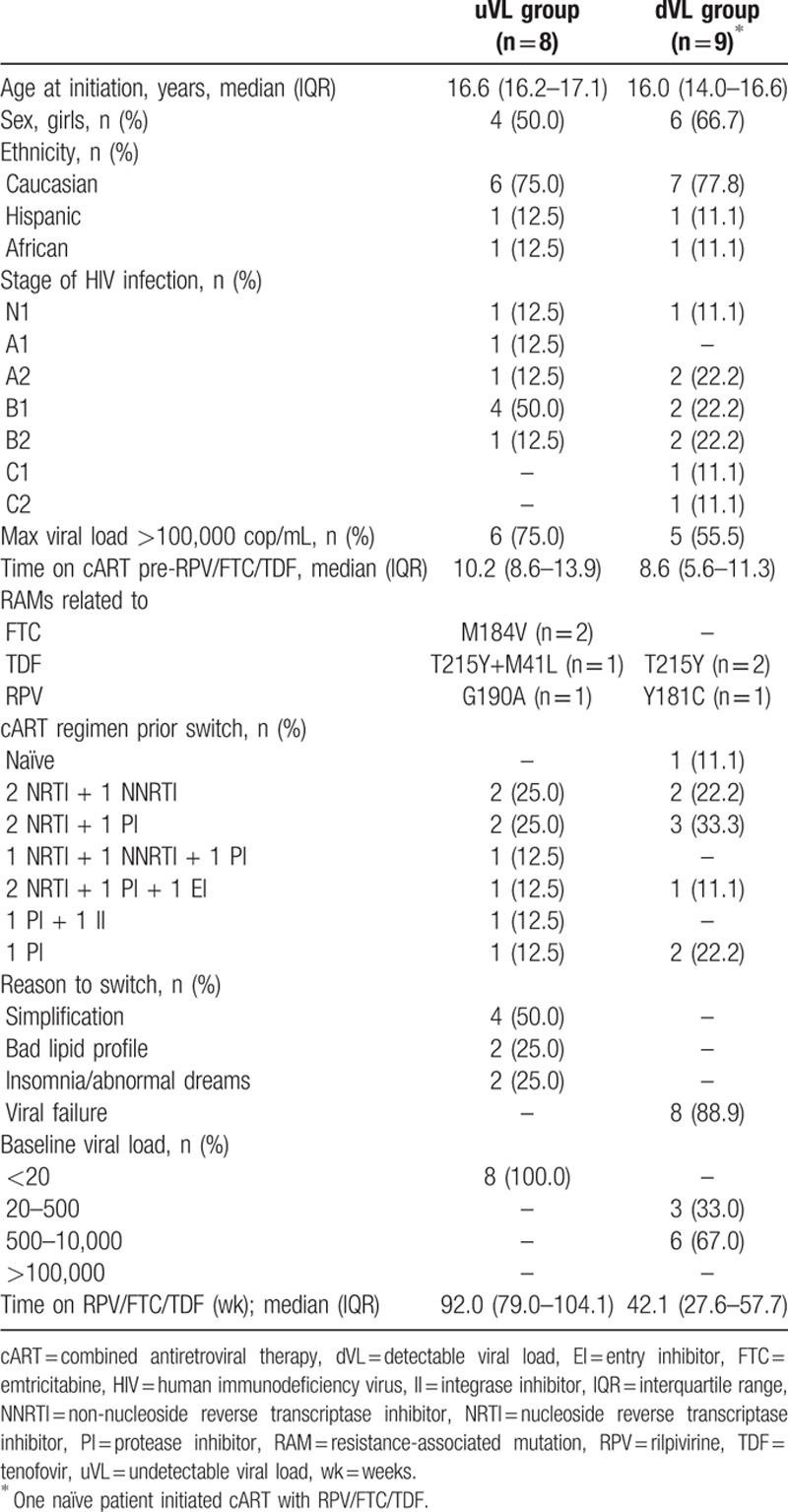
Characteristics of the study population at baseline.

Reasons for RPV/FTC/TDF initiation were simplification (n = 4; 24%); toxicities (neurological, associated with EFV, n = 2, 12%; dyslipidemia, n = 2, 12%); viral failure (n = 8; 47%) and CD4 count below 350/μL in the naïve patient. Overall median time on RPV-based treatment was 61.9 weeks (IQR: 41.1–90.5). According to baseline VL, 8 patients were included in the uVL group and 9 subjects were included in the dVL group. Median time on RPV-based treatment was 89.1 weeks (IQR: 66.5–100.9) and 39.6 weeks (IQR: 23.6–55.4) in the uVL and dVL groups, respectively (*P* = 0.01). Seven out of 8 adolescents with uVL at baseline (including the 3 patients with RAMs) maintained undetectable viral load (uVL) for a median time of 93.6 weeks (IQR: 87.4–104.3). A 17-year-old boy developed viral failure due to poor adherence (<10%) caused by mental disorders (antisocial personality disorder). Eight out of 9 patients in the dVL group achieved and maintained uVL for a total median time of 41.1 weeks (IQR: 26.8–57.5), although 1 of these experienced a blip at the end of the follow-up because of intermediate adherence (50–90%). On the other hand, 1 adolescent remained persistently detectable during the study period because of poor adherence (<70%) and low-level TDF resistance (T215Y), while the patient who had the T215Y and Y181C mutations reached a viral load below 100 copies/mL (77 copies/mL) after 30.9 weeks.

Laboratory parameters are summarized in Table 2. Median CD4 counts as well as CD4/CD8 ratio improved in both groups and a significant difference was observed when analyzing the whole group (*P* = 0.01 and *P* = 0.01, respectively). Five patients changed their immunological category from moderate immunosuppression to no suppression (29%); however, 1 patient dropped from category 1 to 2. No patient discontinued the study because of AEs. Grade 1 AE were reported in 3 patients (18%). Increases in aspartate aminotransferase (AST) and alanine aminotransferase (ALT) occurred in 2 adolescents (12%), although in 1 of them it was not considered related to RPV-based treatment, and triglycerides elevation was reported in 1 patient (6%). While lipid profile in the uVL group improved, it did not reach statistical significance. On the other hand, in the dVL group, cholesterol (*P* = 0.01) improved significantly. Clinical AE related to RPV/FTC/TDF, such as skin rash or central nervous system-related symptoms were not observed during follow-up. Moreover, the 2 adolescents who had suffered insomnia or abnormal dreams with their previous EFV-based regimen did not report any complaints with the new cART. One adolescent with poor adherence (<70%) discontinued the RPV-based regimen and dropped out of care. No deaths were observed.

**Table 2:**
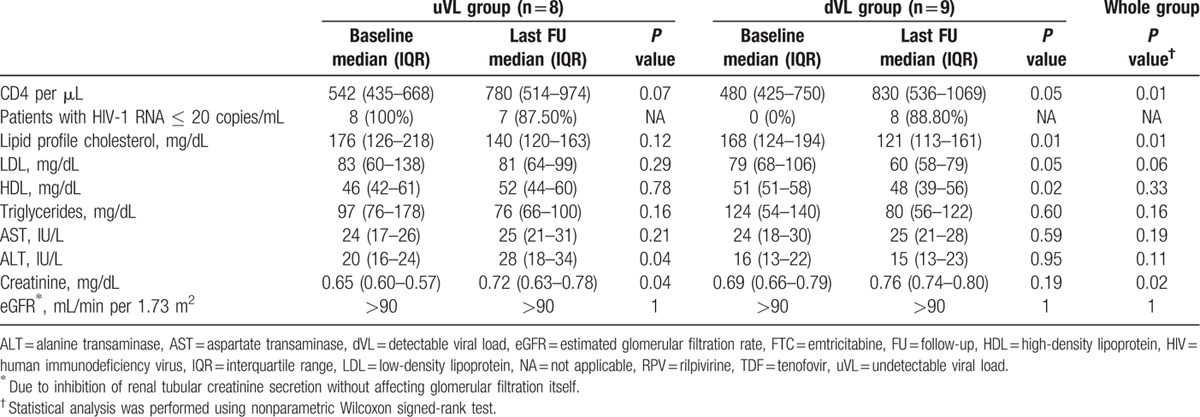
Laboratory parameters at baseline and at last follow-up in pediatric patients treated with RPV/FTC/TDF.

## 3 Discussion

In this case series of HIV-1-infected children and adolescents, treatment with RPV/FTC/TDF was associated with good control of HIV infection in both groups (uVL and dVL) after a median follow-up time of 89.1 and 39.6 weeks, respectively, in all but 2 patients with a poor adherence (<70%). While no clinical or laboratory significant adverse effects were observed, CD4 counts and CD4/CD8 ratio improved independently of the study groups. These observations are in line with previous publications in adults comparing RPV with EFV therapy, which describe a significant increase in CD4 counts after the 96-week treatment period in the RPV group.^[9,10]^ As previously described, it is also worth noting that we observed an improvement of fasting serum lipid profile.^[12]^

Only low-grade AEs were reported during clinical follow-up and the toxicities that lead to cART switch to RPV/FTC/TDF (insomnia/abnormal dreams and bad lipid profile) disappeared. AST, ALT, and creatinine were the commonest observed Grade 3 to 4 laboratory abnormalities described in adults.^[11]^ In this study, Grade 1 AST/ALT and triglycerides elevation were the only laboratory abnormality observed.

In summary, in this first pediatric study, RPV/FTC/TDF was associated with control of viral replication, an improvement of CD4 counts and CD4/CD8 ratio as well as serum lipid profiles, while low-grade AEs were observed. An important limitation of this observational study is the small sample size as well as the different time for follow-up between the 2 groups. However, to date there is limited data on adolescent patients and no data regarding children receiving RPV/FTC/TDF therapy. Our observation is therefore new and probably reassuring, although the presented results need to be confirmed with larger prospective clinical studies in order to conclude whether RPV/FTC/TDF is a good therapeutic option in selected HIV-1-infected children and adolescents where treatment simplification strategies to enhance adherence are crucial.

## 4 Annex

Hospitals in which the patients were enrolled: Hospital Virgen del Rocío, Seville (6 patients); Hospital Universitario Doce de Octubre, Madrid (2 patients); Hospital Universitari Vall d’Hebron, Barcelona (4 patients); Hospital Sant Joan de Déu, Barcelona (3 patients); Hospital General Universitario Gregorio Marañón, Madrid (1 patient); and Hospital Virgen de las Nieves, Granada (1 patient).
